# Chitosan as a sustainable decontamination strategy against *Salmonella* and *Listeria monocytogenes* on pig carcass surfaces: Comparative efficacy with organic acids

**DOI:** 10.14202/vetworld.2026.2554-2567

**Published:** 2026-06-20

**Authors:** Maria Ciríaco, Márcio Moura-Alves, Kamila Soares, Isabel Pinto, Cristina Saraiva, Alexandra Esteves

**Affiliations:** 1 Veterinary and Animal Research Centre (CECAV), University of Trás-os-Montes e Alto Douro (UTAD), Vila Real, Portugal; 2 Associate Laboratory for Animal and Veterinary Sciences (AL4AnimalS), Lisboa, Portugal; 3 Seara SA, Vila Nova de Famalicão, Portugal

**Keywords:** carcass decontamination, chitosan antimicrobial, foodborne pathogens, *Listeria monocytogenes*, meat safety, organic acids, pig carcasses, *Salmonella* control

## Abstract

**Background and Aim:**

Controlling bacterial contamination on pig carcasses is critical for meat safety and public health within the One Health framework. *Salmonella* and *Listeria monocytogenes* are major foodborne pathogens associated with pork products. This study evaluated the decontamination efficacy of chitosan (0.2% and 0.5%) compared to lactic acid (2% and 5%) and citric acid (2% and 5%) against *Salmonella* Typhimurium and *L. monocytogenes* inoculated on pig skin surfaces, while also assessing effects on spoilage microbiota (mesophilic bacteria, psychrotrophic bacteria, and Enterobacteriaceae). The goal was to identify sustainable, natural alternatives to conventional organic acid treatments for early-stage carcass interventions in slaughterhouses.

**Materials and Methods:**

Pig skin samples (25 cm²) were collected from commercial carcasses, inoculated with approximately 5–6 log colony-forming units per square centimeter of the target pathogens or left non-inoculated, and treated by spraying with the respective solutions. Samples were stored at 7 °C for 48 h. Microbial enumeration followed International Organization for Standardization methods for mesophiles (Plate Count Agar, 30 °C/72 h), psychrotrophs (Plate Count Agar, 7 °C/10 days), Enterobacteriaceae (Violet Red Bile Glucose agar), *Salmonella* (Xylose Lysine Deoxycholate agar), and *L. monocytogenes* (Oxford agar). Data were analyzed using one-way analysis of variance and Tukey honestly significant difference test (p < 0.05).

**Results:**

Organic acids provided modest initial reductions (<1 log) in spoilage organisms and mainly bacteriostatic effects against pathogens. Chitosan at 0.5% achieved the strongest reductions, lowering initial mesophilic and psychrotrophic counts by >1 log and maintaining the lowest *L. monocytogenes* levels (only +0.44 log increase over 48 h vs. >2 log in controls). For *Salmonella*, 0.5% chitosan produced a progressive 1.46-log reduction over 48 h (final counts 2.68 log lower than the control), demonstrating bactericidal activity, unlike the bacteriostatic profile of organic acids.

**Conclusion:**

Chitosan, particularly at 0.5%, exhibited superior, more sustained antimicrobial efficacy against both pathogens and spoilage microbiota on pig skin compared with lactic and citric acids. These findings highlight chitosan as a promising natural, sustainable decontamination agent for pig carcasses, with the potential to enhance compliance with European Union microbiological criteria and support greener meat-processing strategies. Further commercial-scale validation and combination approaches are recommended.

## INTRODUCTION

The monitoring and improvement of microbiological characteristics in foods of animal origin contribute to zoonotic disease surveillance within the One Health framework, which emphasizes the interconnectedness of animal health, food production, and public health [1, 2]. Salmonellosis remains one of the most frequently reported zoonoses in the European Union (EU), with 77,485 confirmed human cases, 14,801 hospitalizations, and 88 deaths reported in 2023. Pig meat and pork products have been identified as important sources of human infection [3]. *Listeria monocytogenes* also represents a major public health concern, with 3,145 confirmed cases, 2,500 hospitalizations, and 302 deaths reported during the same period, reflecting its increasing incidence and high case-fatality rate in the EU [3].

Pigs can be asymptomatic carriers of *Salmonella* spp. in their gastrointestinal tract, facilitating the dissemination of this pathogen to carcasses during slaughter operations. In a study conducted at pig slaughterhouses, Costa *et al*. [4] reported a prevalence of 21% of *Salmonella*-positive pig carcasses. The persistence of micro-organisms in asymptomatic animals and their dissemination during transport and lairage have been investigated in slaughterhouse-related studies, which have identified these stages as important points in the contamination pathway [5, 6]. *L. monocytogenes* is more commonly associated with environmental contamination in slaughterhouses and meat processing facilities than with the intestinal tract of pigs. The presence of *L. monocytogenes* on carcass surfaces has been associated with contact with contaminated carcasses, equipment, processing environments, and food handlers, allowing its introduction and persistence along the meat production chain [7–9].

Carcass contamination may occur at multiple stages of the slaughter process, including bleeding, scalding, polishing, evisceration, splitting, and final washing. Studies conducted in slaughterhouse settings have documented microbial contamination during the early stages of the slaughter line, such as bleeding, and have clearly identified the influence of specific processing conditions, including horizontal or recycled-water scalding systems, which have been associated with increased cross-contamination [10, 11]. Polishing and dehairing operations have also been reported to increase contamination with Enterobacteriaceae and *Salmonella* [11]. Evisceration is considered a critical step because accidental rupture of the gastrointestinal tract or contact with contaminated utensils and food handlers may result in direct or indirect carcass contamination [12, 13].

Regulation (EC) No. 2073/2005 establishes process hygiene criteria for *Salmonella* on pig carcasses and includes *L. monocytogenes* as a food safety criterion, highlighting the importance of effective carcass-level control measures [14, 15]. Various physical, chemical, and biological strategies have been investigated to reduce microbial contamination on pig carcass surfaces, with particular emphasis on chemical decontamination using organic acids applied by spraying or misting [16, 17]. Organic acids, such as lactic acid and citric acid, occur naturally in many foods, can be safely incorporated as preservatives, and are considered attractive because of their relatively low toxicity [18–20]. The European Food Safety Authority Panel on Biological Hazards concluded that lactic acid is a safe and effective surface treatment for reducing microbial contamination on carcasses when applied under authorized conditions, with limited concern regarding consumer safety or microbial resistance [21, 22].

In parallel, increasing attention has been paid to natural antimicrobial compounds such as chitosan, a cationic polysaccharide derived primarily from crustacean exoskeletons. Chitosan has demonstrated antimicrobial activity against spoilage and pathogenic micro-organisms, including *L. monocytogenes* and *Salmonella*, particularly in meat preservation systems [23–25]. Its antimicrobial activity has been attributed to interactions with microbial cell membranes, alterations in membrane permeability, and interference with essential cellular functions, making it a promising alternative to conventional chemical decontaminants.

Despite growing interest in chitosan as a natural antimicrobial agent, most studies to date have focused on its use in post-packaging interventions, including edible coatings, active films, antimicrobial packaging systems, and preservation treatments during storage and distribution. Comparatively limited information is available regarding its direct application as a carcass decontamination treatment immediately after slaughter, when microbial contamination can be controlled at critical points before further processing. Moreover, few studies have directly compared the efficacy of chitosan with that of commonly used organic acids under identical experimental conditions on pig carcass surfaces. The effectiveness of decontamination treatments may vary substantially depending on the food matrix, microbial species, contamination level, and application method. Consequently, there remains a lack of matrix-specific evidence regarding chitosan’s ability to control foodborne pathogens on porcine carcass surfaces and its potential role as a sustainable intervention in modern pork production systems.

Therefore, this study aimed to evaluate and compare the decontamination efficacy of chitosan and organic acids against *Salmonella* Typhimurium and *L. monocytogenes* inoculated onto pig skin surfaces. In addition, the effects of these treatments on hygiene and spoilage indicator micro-organisms, including mesophilic bacteria, psychrotrophic bacteria, and Enterobacteriaceae, were assessed during refrigerated storage. By generating matrix-specific data under controlled experimental conditions, this study sought to assess the potential of chitosan as a sustainable carcass decontamination strategy and to provide evidence to support interventions targeting critical control points for pathogen transmission within the pork production chain.

## MATERIALS AND METHODS

### Ethical approval

This study was conducted using pig skin samples collected from commercial crossbred fattening pigs after routine slaughter procedures at an authorized industrial abattoir in northern Portugal. No live animals were subjected to experimental procedures, handling, restraint, treatment, or euthanasia specifically for the purposes of this research. All animals included in the study were slaughtered as part of standard commercial meat production practices in accordance with applicable EU animal welfare legislation and slaughterhouse regulations.

Postmortem pig skin samples were collected after completion of the slaughter process and did not interfere with animal welfare, slaughter operations, or commercial processing activities. Therefore, the study did not involve the experimental use of live animals and was exempt from formal approval for animal experimentation under institutional and national guidelines governing the ethical use of animals in research.

Permission to collect samples was obtained from the management of the participating slaughterhouse. All laboratory procedures involving bacterial pathogens were conducted in accordance with institutional biosafety requirements and standard microbiological safety protocols to ensure the safe handling, processing, and disposal of biological materials.

### Study period and location

The study was conducted using pig skin samples obtained from commercial crossbred fattening pigs slaughtered at 5–6 months of age at an industrial abattoir located in northern Portugal (41°24′50.2″ N, 8°29′49.9″ W). Laboratory procedures, including inoculation, decontamination treatments, microbiological analyses, and storage experiments, were performed under controlled laboratory conditions. Samples were analyzed at predefined storage intervals of 0, 6, 12, 24, and 48 h.

### Study design

**Figure 1:**
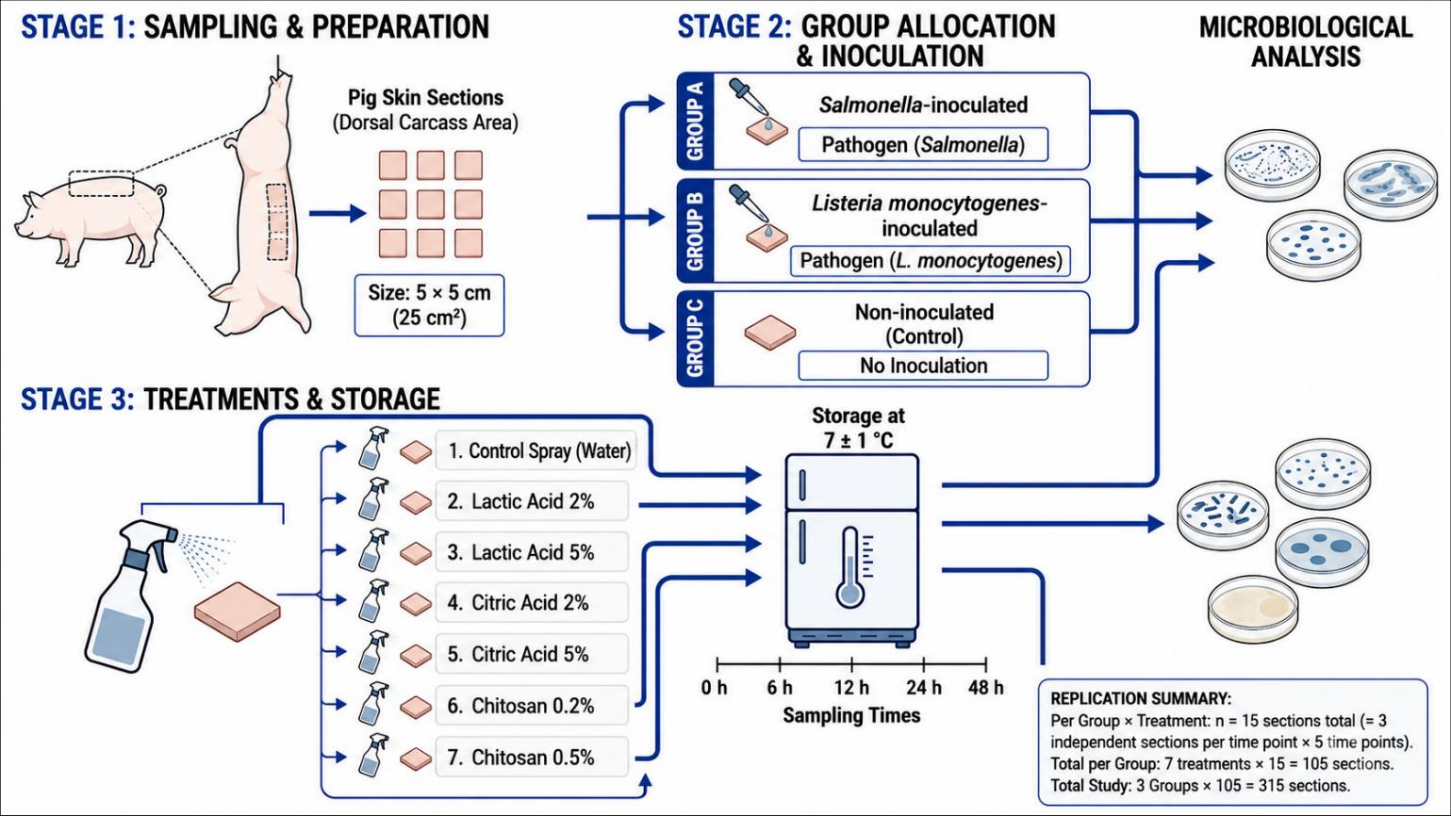
Experimental design of the study. Created using SciDraw® software (https://sci-draw.com).

The graphical representation of the experimental workflow is presented in Figure 1. The diagram summarizes the study design, including pig skin sample collection, group allocation, inoculation procedures, application of decontamination treatments, storage conditions, and subsequent microbiological analyses.

A completely randomized experimental design with destructive sampling was employed. Pig skin sections were excised from the dorsal region of carcasses after dressing and cut into standardized 25 cm² (5 × 5 cm) sections. Samples were randomly assigned to treatment groups and storage periods. Independent skin sections were analyzed at each sampling time point (0, 6, 12, 24, and 48 h).

Samples were divided into three experimental groups: samples inoculated with *Salmonella enterica* subsp. *enterica*, samples inoculated with *L. monocytogenes*, and non-inoculated samples. Non-inoculated samples were used to enumerate hygiene and spoilage indicator micro-organisms, including total aerobic mesophilic bacteria, psychrotrophic bacteria, and Enterobacteriaceae, as well as to detect *L. monocytogenes* and *Salmonella* spp. In inoculated samples, the respective pathogens (*S. enterica* subsp. *enterica* or *L. monocytogenes*) were enumerated to evaluate the decontamination efficacy of the treatments.

All samples, irrespective of inoculation status, were treated with lactic acid (2% and 5%, v/v), citric acid (2% and 5%, w/v), or chitosan (0.2% and 0.5%, w/v), and included an untreated control group. The distribution of samples among treatments and inoculation groups is presented in Table 1.

**Table 1:** Experimental design and distribution of pig skin samples according to treatment and inoculation status. Values represent the number of independent pig skin sections (n) analyzed per treatment.

Treatments	*Salmonella*	*L. monocytogenes*	Not inoculated
Control	15	15	15
Lactic acid 2%	15	15	15
Lactic acid 5%	15	15	15
Citric acid 2%	15	15	15
Citric acid 5%	15	15	15
Chitosan 0.2%	15	15	15
Chitosan 0.5%	15	15	15
Total (n)	105	105	105

### Inoculum preparation

A combination of *Salmonella* strains was prepared using *S*. Typhimurium American Type Culture Collection (ATCC) 14028 (collection strain) and *Salmonella Derby* isolated from a slaughterhouse. The *L. monocytogenes* inoculum consisted of a mixture of *L. monocytogenes* ATCC 7973 (collection strain) and *L. monocytogenes* isolated from slaughterhouse facilities.

The cultures were preserved at −20°C in Brain Heart Infusion (BHI) medium (VWR Chemicals, Mumbai, India) supplemented with 25% (v/v) glycerol. Bacterial strains were initially subcultured in BHI medium and incubated at 37°C for 24 h. Following incubation, bacterial suspensions were streaked onto Xylose Lysine Deoxycholate agar (VWR Chemicals, Mumbai, India) and incubated at 37°C for an additional 24 h. Isolated colonies were subsequently transferred into BHI medium and incubated at 37°C for a further 24 h.

Suspensions of each bacterial strain were obtained by centrifugation at 10,000 × *g* for 10 min at 4°C using a Sigma 3k18 centrifuge (Sigma Laborzentrifugen GmbH, Osterode am Harz, Germany). The supernatant was discarded, and the pellet was resuspended in sterile 0.9% sodium chloride solution. This washing procedure was repeated three times. Standardized bacterial concentrations were determined using calibration curves based on optical density measurements at 600 nm. Subsequently, bacterial strains were combined in equal proportions (1:1) and subjected to serial decimal dilutions (1:10) in sterile 0.9% sodium chloride solution.

The concentration of *Salmonella* was confirmed on Xylose Lysine Deoxycholate agar (VWR Chemicals) following incubation at 37°C for 24 h. The concentration of *L. monocytogenes* was confirmed on Oxford Listeria agar (Liofilchem, Roseto degli Abruzzi, Italy).

### Inoculation procedure

**Figure 2:**
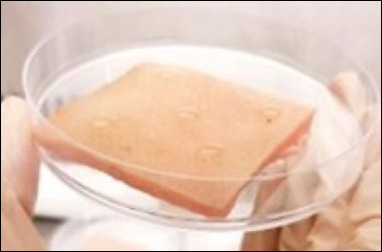
Inoculation surface showing five inoculation points.

Samples were placed in sterile Petri dishes and inoculated with 100 μL of bacterial suspension at five distinct locations, as illustrated in Figure 2. The resulting inoculum concentrations were 4.92 × 10^6^ colony-forming units (CFU)/cm² for *Salmonella* and 6.38 × 10^6^ CFU/cm² for *L. monocytogenes*.

To ensure homogeneous coverage, the inoculum was distributed across the entire sample surface using a sterile L-shaped spreader for approximately 10 s per sample. Subsequently, samples were air-dried in a laminar flow cabinet for 30 min to facilitate bacterial attachment. Viable counts were verified immediately after inoculation (0 h) by plating appropriate dilutions to confirm effective contamination and the consistency of the inoculum level.

### Decontamination procedure and storage

Lactic acid (90% solution; VWR Chemicals, Belgium) was diluted with sterile distilled water to obtain final concentrations of 2% and 5% (v/v). Citric acid (≥99% purity; VWR Chemicals, Belgium) was dissolved in sterile distilled water to prepare 2% and 5% (w/v) solutions.

Chitosan (50–190 kDa; Sigma-Aldrich Chemie, Riedstr, Germany), with a degree of deacetylation of 75%, was dissolved in 1% (v/v) glacial acetic acid under continuous stirring for 24 h at room temperature to prepare 0.2% and 0.5% (w/v) solutions. Following complete dissolution, the pH of the chitosan solutions was adjusted to 4.5–5.0 using 1 M sodium hydroxide solution (VWR, Sweden). The pH was measured using a calibrated digital pH meter (WTW pH330i; WTW, Weinheim, Germany).

Following inoculation, approximately 0.6 mL of each treatment solution was applied to the sample surface by spraying. A commercial spray bottle was mounted on a support positioned 15 cm above the samples, which were placed on a flat surface to ensure uniform application. All treatments were applied at room temperature. The spraying distance was maintained constant throughout the experiment to ensure consistency and reproducibility among treatments.

Excess treatment solution was retained within the Petri dishes throughout the experimental period to prevent drainage-mediated removal of inoculated bacteria and consequent underestimation of surface microbial counts.

All samples were stored at 7°C ± 1°C in a laboratory refrigerator monitored using a data logger (Testo 174; Testo SE & Co. KGaA, Titisee-Neustadt, Germany) and analyzed after 0, 6, 12, 24, and 48 h of storage.

### Microbiological analysis

Microbiological determinations were performed according to International Organization for Standardization (ISO) methodologies with minor procedural adaptations to accommodate the experimental design.

Samples were diluted in 40 mL of Tryptone Salt (Himedia, Mumbai, India) and homogenized using a Stomacher for 60 s. To ensure complete microbial recovery, the internal surfaces of the 90-mm Petri dishes were rinsed with a portion of the diluent used for sample preparation, thereby ensuring that the entire microbial load was included in the analysis. Serial decimal dilutions were subsequently prepared.

For non-inoculated samples, the presence of *Salmonella* spp. and *L. monocytogenes* was investigated using ISO-specified detection methods. Detection of *Salmonella* spp. was performed following ISO 6579-1 [26], including pre-enrichment in Buffered Peptone Water, selective enrichment, and plating on Xylose Lysine Deoxycholate agar (XLD; VWR Chemicals, Mumbai, India), followed by incubation at 37°C for 24 h. Presumptive colonies were confirmed according to the ISO protocol.

Detection of *L. monocytogenes* was performed according to ISO 11290-1 [27], including selective enrichment and plating on Oxford Listeria agar (Liofilchem, Roseto degli Abruzzi, Italy), followed by incubation at 37°C for 24–48 h and confirmation of characteristic colonies.

Total aerobic mesophilic bacteria were enumerated using Plate Count Agar (PCA; Liofilchem, Teramo, Italy) after incubation at 30°C for 72 h, in accordance with ISO 4833-1 [28]. Psychrotrophic bacteria were enumerated on PCA after 10 days of incubation at 7°C, in accordance with ISO 17410 [29]. Enterobacteriaceae were enumerated on Violet Red Bile Glucose agar (VRBG; Liofilchem, Teramo, Italy) after incubation at 37°C for 24 h, in accordance with ISO 21528-2 [30].

*Salmonella* was enumerated on XLD agar after incubation at 37°C for 24 h, in accordance with ISO 6579-1 [26]. *L. monocytogenes* was enumerated on Oxford Listeria agar following incubation at 37°C for 24–48 h according to the principles of ISO 11290-2 [31], with adaptation of the selective medium.

Results were expressed as log_10_ colony-forming units per square centimeter (log CFU/cm²). The detection limit of the enumeration methods was 1.0 log CFU/cm². Counts below this threshold were recorded as <1.0 log CFU/cm² and assigned a value of 0 log CFU/cm² for statistical analyses.

### Statistical analysis

Results were expressed as mean microbial counts ± standard deviation (log CFU/cm²). Statistical analyses were performed using Statistica version 12 (StatSoft, Tulsa, OK, USA). Data normality was assessed using the Shapiro–Wilk test. Differences among treatments and storage periods were evaluated using one-way analysis of variance. Mean comparisons were performed using Tukey’s honestly significant difference test. Statistical significance was established at p < 0.05.

## RESULTS

### Effects of treatments on hygiene and spoilage indicator micro-organisms

Tables 2–4 present the counts of mesophilic bacteria, psychrotrophic bacteria, and Enterobacteriaceae, respectively, in non-inoculated samples after the application of organic acids and chitosan during 48 h of storage.

**Table 2:** Counts of mesophilic bacteria (log CFU/cm², mean ± standard deviation) over 48 h after decontamination with organic acids and chitosan and the respective control.

Treatment	0 h	6 h	12 h	24 h	48 h	Effect
Control	5.87 ± 0.09^cd^	5.68 ± 0.13d^AB^	6.12 ± 0.02^cAB^	7.78 ± 0.01^b^	8.30 ± 0.04^a^	***
Lactic acid 2%	5.73 ± 0.09^cd^	5.32 ± 0.17d^AB^	6.16 ± 0.09^cAB^	7.43 ± 0.19^b^	8.12 ± 0.21^a^	***
Lactic acid 5%	4.77 ± 0.08^b^	4.84 ± 0.43^bB^	5.77 ± 0.20^bB^	6.91 ± 0.50^a^	7.98 ± 0.60^a^	***
Citric acid 2%	5.09 ± 0.19^d^	5.69 ± 0.15^cdAB^	6.44 ± 0.33^bcAB^	7.23 ± 1.10^ab^	7.97 ± 0.56^a^	**
Citric acid 5%	4.79 ± 0.15^c^	5.21 ± 0.73^cAB^	5.81 ± 0.16^bcAB^	6.69 ± 0.17^ab^	7.68 ± 0.04^a^	***
Chitosan 0.2%	5.48 ± 0.30^d^	6.09 ± 0.18^cdA^	6.52 ± 0.52^bcA^	7.26 ± 0.23^b^	8.71 ± 0.03^a^	***
Chitosan 0.5%	4.64 ± 1.19^b^	5.69 ± 0.28^bAB^	5.87 ± 0.06^bAB^	7.46 ± 0.14^a^	8.09 ± 0.22^a^	***
Effect	n.s.	*	*	n.s.	n.s.	

n.s. = Non-significant (p ≥ 0.05). For storage time (rows), means with different lowercase letters differ significantly. For decontamination treatment (columns), means with different uppercase letters differ significantly.

* p < 0.05,

** p < 0.01,

*** p < 0.001.

**Table 3:** Counts of psychrotrophic bacteria (log CFU/cm², mean ± standard deviation) over 48 h after decontamination with organic acids and chitosan and the respective control.

Treatment	0 h	6 h	12 h	24 h	48 h	Effect
Control	4.10 ± 0.02^dA^	4.01 ± 0.06^dAB^	5.22 ± 0.08^cA^	5.53 ± 0.05^b^	6.19 ± 0.11^a^	***
Lactic acid 2%	3.48 ± 0.25^dAB^	3.46 ± 0.22^dAB^	4.23 ± 0.10^cB^	5.52 ± 0.20^b^	6.42 ± 0.10^a^	***
Lactic acid 5%	2.84 ± 0.22^dB^	3.05 ± 0.39^cdB^	3.79 ± 0.19^cB^	4.88 ± 0.39^b^	6.27 ± 0.32^a^	***
Citric acid 2%	3.31 ± 0.45^bAB^	3.83 ± 0.07^bAB^	4.12 ± 0.10^abB^	5.50 ± 0.85^a^	6.42 ± 0.50^a^	**
Citric acid 5%	2.88 ± 0.13^cAB^	3.22 ± 0.81^cAB^	3.91 ± 0.15^bcB^	4.76 ± 0.30^b^	6.10 ± 0.03^a^	***
Chitosan 0.2%	3.54 ± 0.10^dAB^	4.28 ± 0.15^cA^	4.38 ± 0.25^cB^	5.46 ± 0.36^b^	6.86 ± 0.03^a^	***
Chitosan 0.5%	2.52 ± 0.81^cB^	3.80 ± 0.31^bAB^	4.11 ± 0.11^bB^	5.72 ± 0.31^a^	6.42 ± 0.30^a^	***
Effect	**	*	***	n.s.	n.s.	

n.s. = Non-significant (p ≥ 0.05). For storage time (rows), means with different lowercase letters differ significantly. For decontamination treatment (columns), means with different uppercase letters differ significantly.

* p < 0.05,

** p < 0.01,

*** p < 0.001.

**Table 4:** Counts of Enterobacteriaceae (log CFU/cm², mean ± standard deviation) over 48 h after decontamination with organic acids and chitosan and the respective control.

Treatment	0 h	6 h	12 h	24 h	48 h	Effect
Control	4.29 ± 0.03^c^	4.03 ± 0.06^AcA^	5.14 ± 0.01^bA^	5.51 ± 0.34^bA^	6.62 ± 0.18^a^	***
Lactic acid 2%	3.36 ± 0.15^b^	2.46 ± 0.36^cB^	3.49 ± 0.09^bBC^	4.97 ± 0.29^aAB^	5.72 ± 0.41^a^	***
Lactic acid 5%	2.89 ± 0.25^b^	2.97 ± 0.56^bAB^	2.87 ± 0.27^bC^	4.60 ± 0.57^aAB^	5.85 ± 0.73^a^	***
Citric acid 2%	3.08 ± 0.62^b^	3.36 ± 0.33^bAB^	3.44 ± 0.29^bBC^	4.82 ± 0.15^aAB^	5.34 ± 0.76^a^	***
Citric acid 5%	2.99 ± 0.57^bc^	2.35 ± 0.43^cB^	3.31 ± 0.35^bcBC^	4.05 ± 0.37^abB^	4.81 ± 0.31^a^	***
Chitosan 0.2%	2.84 ± 0.73^c^	3.37 ± 0.35^cAB^	3.96 ± 0.73^bcB^	4.99 ± 0.18^abAB^	6.38 ± 0.43^a^	***
Chitosan 0.5%	2.92 ± 0.17^b^	2.74 ± 0.54^bAB^	2.99 ± 0.08^bBC^	4.91 ± 0.41^aAB^	4.96 ± 0.69^a^	**
Effect	n.s.	*	**	*	n.s.	

n.s. = Non-significant (p ≥ 0.05). For storage time (rows), means with different lowercase letters differ significantly. For decontamination treatment (columns), means with different uppercase letters differ significantly.

* p < 0.05,

** p < 0.01,

*** p < 0.001.

For mesophilic bacteria, chitosan at 0.5% exhibited the lowest initial counts at 0 h, with reductions exceeding 1 log CFU/cm² relative to the control. However, after 6 h, both chitosan concentrations showed bacterial counts similar to those of the control during storage. After 48 h, the lowest mesophilic counts were observed in samples treated with 5% citric acid, with no significant differences among treatments at this time point.

For psychrotrophic bacteria, treatment with 0.5% chitosan led to reductions of approximately 1.5 log CFU/cm² at 0 h (p < 0.01) and maintained levels approximately 1 log CFU/cm² lower than the control up to 12 h (p < 0.001). Treatments with lactic acid and citric acid, particularly at 5%, consistently resulted in lower psychrotrophic counts than the control during the first 24 h. By the end of storage, psychrotrophic populations increased in all treatments, reaching levels comparable to the control, with no significant differences between groups.

For Enterobacteriaceae, both chitosan concentrations (0.2% and 0.5%) produced initial reductions exceeding 1 log CFU/cm² relative to the control. After 48 h, samples treated with chitosan showed counts approximately 1.6–1.8 log CFU/cm² lower than the control, mainly reflecting the increase observed in the untreated control during storage. Organic acid treatments also resulted in significant initial reductions, with 5% citric acid maintaining lower counts than the control and chitosan groups after 24 h. By 48 h, these differences decreased, with counts approaching the results obtained for samples treated with 0.5% chitosan.

### Effects of treatments on *Salmonella*

Table 5 presents *Salmonella* counts for inoculated samples treated with chitosan and organic acids during 48 h of storage, along with the respective controls.

**Table 5:** Counts of *Salmonella* (log CFU/cm², mean ± standard deviation) over 48 h after decontamination with organic acids and chitosan.

Treatment	0 h	6 h	12 h	24 h	48 h	Effect
Control	5.48 ± 0.07^a^	5.67 ± 0.14^a^	6.51 ± 0.08^bB^	6.57 ± 0.20^bD^	6.97 ± 0.04^cA^	***
Lactic acid 2%	5.14 ± 0.24^a^	5.14 ± 0.75^a^	5.56 ± 0.31^abA^	5.56 ± 0.25^abBC^	6.64 ± 0.27^bA^	**
Lactic acid 5%	5.12 ± 0.16	5.10 ± 0.62	5.17 ± 0.42^A^	5.07 ± 0.16^AB^	5.35 ± 0.32^BC^	n.s.
Citric acid 2%	5.29 ± 0.20^a^	6.01 ± 0.17^ab^	5.90 ± 0.28^abAB^	5.83 ± 0.22^abC^	6.52 ± 0.53^bA^	**
Citric acid 5%	5.56 ± 0.94	5.78 ± 0.11	5.37 ± 0.53^A^	5.47 ± 0.07^ABC^	5.98 ± 0.19^AC^	n.s.
Chitosan 0.2%	5.36 ± 0.77	5.68 ± 0.49	5.54 ± 0.18^A^	5.24 ± 0.07^ABC^	4.91 ± 1.10^BC^	n.s.
Chitosan 0.5%	5.75 ± 0.18^b^	5.69 ± 0.21^ab^	5.50 ± 0.18^abA^	5.24 ± 0.07^abA^	4.29 ± 0.45^aB^	*
Effect	n.s.	n.s.	**	***	***	

n.s. = Non-significant (p ≥ 0.05). For storage time (rows), means with different lowercase letters differ significantly. For decontamination treatment (columns), means with different uppercase letters differ significantly.

* p < 0.05,

** p < 0.01,

*** p < 0.001.

In control samples, a highly significant increase in *Salmonella* counts was observed over the storage period (p < 0.001), corresponding to a 1.49 log CFU/cm² increase, from 5.48 to 6.97 log CFU/cm². The greatest increase was observed between 6 and 12 h of storage (0.84 log CFU/cm²).

An increase in *Salmonella* counts was observed during storage in samples treated with lactic acid and citric acid. Counts increased by 1.50 and 0.23 log CFU/cm² for 2% and 5% lactic acid, respectively, and by 1.23 and 0.42 log CFU/cm² for 2% and 5% citric acid, respectively. In contrast, samples treated with chitosan at 0.2% and 0.5% showed changes of −0.45 and −1.46 log CFU/cm² over 48 h, respectively, with a statistically significant difference at 0.5% (p < 0.05). After 48 h, *Salmonella* counts in the 0.5% chitosan group were 2.68 log CFU/cm² lower than those in the control.

### Effects of treatments on *L. monocytogenes*

Table 6 presents *L. monocytogenes* counts for inoculated samples treated with chitosan and organic acids during 48 h of storage, along with the corresponding controls.

**Table 6:** Counts of *Listeria monocytogenes* (log CFU/cm², mean ± standard deviation) during 48 h of storage after decontamination with organic acids and chitosan.

Treatment	0 h	6 h	12 h	24 h	48 h	Effect
Control	5.96 ± 0.28^aB^	6.04 ± 0.81^aD^	5.64 ± 0.23^aB^	6.36 ± 0.17^aB^	8.15 ± 0.70^bB^	***
Lactic acid 2%	4.49 ± 0.46^aAB^	4.54 ± 0.47^aA^	4.61 ± 0.04^abA^	5.62 ± 0.19^bAB^	6.92 ± 0.51^cA^	***
Lactic acid 5%	4.68 ± 0.72^aA^	4.22 ± 0.40^aA^	4.66 ± 0.87^aA^	5.43 ± 0.42^abAB^	6.63 ± 0.03^bA^	**
Citric acid 2%	4.87 ± 0.42^abA^	4.20 ± 0.68^aBC^	5.10 ± 0.24^abAB^	5.65 ± 0.35^bAB^	7.17 ± 0.66^cA^	***
Citric acid 5%	5.02 ± 0.11^aAB^	4.87 ± 0.70^aCD^	4.84 ± 0.44^aA^	5.20 ± 0.21^aAB^	7.27 ± 0.29^bAB^	***
Chitosan 0.2%	5.00 ± 0.21^abAB^	4.90 ± 0.58^aBC^	4.41 ± 0.26^aA^	5.22 ± 0.12^abA^	5.92 ± 0.36^bA^	**
Chitosan 0.5%	5.05 ± 0.03^aAB^	4.94 ± 0.25^abAB^	4.39 ± 0.29^bA^	5.11 ± 0.06^aAB^	5.49 ± 0.29^aA^	**
Effect	**	***	**	*	***	

n.s. = Non-significant (p ≥ 0.05). For storage time (rows), means with different lowercase letters differ significantly. For decontamination treatment (columns), means with different uppercase letters differ significantly.

* p < 0.05,

** p < 0.01,

*** p < 0.001.

For control samples, counts remained relatively stable during the first 12 h, ranging from 5.96 ± 0.28 to 5.64 ± 0.23 log CFU/cm², followed by a progressive increase to 8.15 ± 0.70 log CFU/cm² after 48 h (p < 0.001), corresponding to an increase of 2.19 log CFU/cm² over 48 h.

Samples treated with chitosan exhibited lower counts than the control throughout the storage period for both concentrations tested. During the first 24 h, counts remained between 5.00 and 5.22 log CFU/cm², showing only a modest increase. After 48 h, the 0.5% chitosan treatment maintained the lowest counts among all treatments, increasing from 5.05 to 5.49 log CFU/cm², corresponding to a limited increase of 0.44 log CFU/cm² over 48 h. This represents a difference of 2.66 log CFU/cm² compared with the control.

For samples treated with organic acids, both lactic acid and citric acid initially reduced microbial counts compared with the control, although significant increases were observed over time. After 48 h, 5% citric acid and 5% lactic acid showed increases of 2.25 and 1.95 log CFU/cm², respectively, but still maintained lower counts than the control by 0.88 and 1.52 log CFU/cm², respectively.

## DISCUSSION

### Influence of chitosan and organic acids on spoilage micro-organism counts

Treated and control samples demonstrated a significant increase in microbial populations throughout the storage period. A potential initial inhibitory effect on spoilage micro-organisms was observed, as indicated by lower counts at 0 h after treatment with 0.5% chitosan, 5% citric acid, and 5% lactic acid, mainly for psychrotrophic micro-organisms (p < 0.01).

Under refrigerated storage, psychrotrophic bacteria become dominant members of the spoilage microbiota in meat systems [32]. However, adaptation to low temperature and acid tolerance involves distinct regulatory responses [33]. Because many psychrotrophic spoilage bacteria in chilled meat are Gram-negative and chitosan has been shown to interact with negatively charged bacterial membranes, altering membrane permeability [34, 35], such membrane-targeting mechanisms may have contributed to the reductions observed in the present study. In contrast, mesophilic counts may reflect a broader and more heterogeneous fraction of the resident microbiota, encompassing taxa with variable physiological responses to environmental stress. The subsequent recovery of mesophilic counts is consistent with ecological succession and regrowth dynamics described for chilled meat systems [32].

The scientific literature currently lacks extensive studies on the direct application of chitosan to whole porcine carcasses. However, research on other carcasses and meat products consistently confirms its strong potential as a biopreservative. Ramezani *et al*. [36] reported that treating quail carcasses with 1% (w/v) lactic acid and chitosan combined with modified atmosphere packaging (MAP, 65% CO_2_, 30% N_2_, and 5% O_2_) extended carcass shelf life. For total viable counts, treated samples had 0.5–3.4 log CFU/g lower counts than control samples on day 8 of storage. The weakest effect was produced by lactic acid alone, whereas the strongest effect was produced by the combination of lactic acid, chitosan, and MAP. In another study, Vasilatos and Savvaidis [37] achieved an 8-day shelf-life extension for turkey meat treated by dipping it in a 1.5% (w/v) chitosan solution, followed by vacuum packaging and storage at 2°C. Petrou *et al*. [38] reported an extended shelf life of chicken breast meat treated with 1.5% (w/v) chitosan in combination with a modified atmosphere containing 30% CO_2_, and 70% NO_2_, with samples stored at 4°C.

The results obtained after application of organic acids are comparable with previous findings. Sallam *et al*. [39] evaluated the efficacy of 2% lactic acid, 2% acetic acid, and 12% trisodium phosphate against aerobic bacteria, Enterobacteriaceae, enterococci, coliforms, and *Staphylococcus aureus* in 25 swine carcass samples. The application of 2% lactic acid to shoulder samples resulted in reductions of 1.38 log for aerobic bacteria and 1.95 log for Enterobacteriaceae. In thigh samples, the observed reductions were 1.62 log and 1.76 log for the same microbial groups, respectively. Application of 2% acetic acid resulted in reductions of 1.46 and 1.36 log for aerobic micro-organisms in shoulder and thigh samples, respectively, and reductions of 2.22 and 1.64 log for Enterobacteriaceae in the corresponding samples. In the present study, reductions achieved with 2% lactic acid or 2% citric acid were less than 1 log. Reductions above 1 log were obtained only with 5% lactic acid, 5% citric acid, and chitosan. These differences may be explained by variation in initial contamination load and the specific carcass areas sampled, factors known to strongly influence the effectiveness of decontamination treatments. Recent insights provided by Sorathiya *et al*. [20] reinforce that the efficacy of organic acids is strongly influenced by their degree of dissociation, pKa, food matrix buffering capacity, and environmental pH, factors that may explain the different results observed in the present study when acids were applied to pork skin [20].

Although direct studies specifically focusing on pig skin decontamination remain limited, recent research on pork matrices demonstrates that antimicrobial efficacy is influenced by the characteristics of the porcine substrate. Zhao *et al*. [40] reported that treatments with chitosan, nisin, and tea polyphenols applied to fresh chilled pork primarily delayed microbial growth rather than causing immediate inactivation, indicating matrix-dependent modulation of antimicrobial effectiveness. Similarly, Kalita *et al*. [41] observed that chitosan-based coatings applied to pork meat exerted a progressive antimicrobial effect during refrigerated storage, acting mainly as a sustained protective barrier. Zhang *et al*. [42] further demonstrated that chitosan-based coatings on pork slices significantly delayed microbial proliferation over time.

### Influence of chitosan and organic acids on *Salmonella* counts

Previous studies evaluated the effectiveness of chitosan at a high concentration (2%) against *S. enterica* inoculated at 5 log CFU/g onto turkey fillets, which were stored at 4°C and 10°C for 21 days. A highly significant reduction in *S. enterica* was observed in samples stored at 10°C on day 21 of storage [43]. In another study, 0.5%, 1%, and 2% chitosan, 1% lactic acid, and the combination of 2% chitosan and 1% lactic acid were used to control *S*. Typhimurium and *Escherichia coli* in chicken fillets stored at 4°C. Treatment with 0.5% chitosan did not reduce *S*. Typhimurium or *E. coli* counts. *S*. Typhimurium was reduced only by 2% chitosan, 1% lactic acid, and the combination of 2% chitosan and 1% lactic acid, with reductions of approximately 1.5 log [44]. Kaplan *et al*. [45] inoculated broiler carcasses with 10^8^ CFU/mL of *S*. Typhimurium to evaluate the effect of immersion for 5 min in 2% lactic acid and 0.1% chitosan solutions. *Salmonella* counts were determined at 0, 3, and 7 days after treatment. Reductions of 1.5 CFU/mL and 1.66 CFU/mL were observed for lactic acid and chitosan treatments, respectively [45].

In the present study, the application of 0.2% and 0.5% chitosan resulted in a distinct trend compared with the organic acids tested. Although 5% lactic acid and 5% citric acid generally exhibited a bacteriostatic effect, with non-significant reductions over time, chitosan, particularly at 0.5%, produced a progressive decrease in *Salmonella* counts, reaching a statistically significant reduction after 48 h (p < 0.05). This behavior suggests that, under the tested conditions, chitosan exerted a concentration-dependent inhibitory effect.

Dan *et al*. [46] reported reductions of approximately 2.3 log CFU/g after 30 min of treatment with 3% lactic acid and citric acid in beef inoculated with *Salmonella*. In another study using chicken meat samples, *S*. Typhimurium ATCC 14028 was inoculated at two levels (10² and 10^8^ CFU/g) and subsequently decontaminated by immersion in baths containing 1% lactic acid and citric acid. For both inoculation levels, a reduction in *Salmonella* populations was observed 30 min after treatment. In samples inoculated with 10^8^ CFU/g, reductions of 88% and 72% were specifically reported for lactic acid and citric acid treatments, respectively [47]. In another study conducted on bovine carcasses, post-slaughter spraying with 3% lactic acid and 3% acetic acid resulted in a reduction of 2.66 log CFU/10 cm² in *S. enterica* subsp. *enterica* after 24 h in the lactic acid-treated group [17].

In the present study, in which 5% lactic acid and 5% citric acid solutions were applied, *Salmonella* counts showed no significant variation over time (p > 0.05), suggesting a bacteriostatic effect attributable to the decontaminating agents tested. These results are consistent with the findings of Yeh *et al*. [48], who reported that, under similar experimental conditions, application of lactic acid to ground meat samples inoculated with a mixture of *Salmonella* spp. was not associated with a significant reduction in *Salmonella* counts [48].

The differences observed between the present study and previous reports may reflect variations in the treated matrix, particularly the use of pig skin in the present work, as well as differences in application method, antimicrobial concentration, exposure time, and initial contamination level. In addition, Yoon *et al*. [49] emphasized that the effectiveness of organic acids against foodborne pathogens varies widely depending on factors such as concentration, medium pH, and exposure time. These authors reported that, in many experimental scenarios, a bacteriostatic rather than an immediate bactericidal effect is more frequently observed, which supports the results obtained in the present study. They also highlighted that the food matrix can protect micro-organisms from acid activity, explaining why results obtained in real meat systems are often less pronounced than those obtained in laboratory media, and noted that higher initial contamination levels reduce the effectiveness of organic acids [49]. In this context, Sorathiya *et al*. [20] further suggested that combining organic acids with other natural antimicrobials, such as essential oils or biopolymers such as chitosan, may result in synergistic effects [20]. Based on the present findings, this approach could be particularly promising because chitosan showed gradual but effective antimicrobial activity, which may complement the rapid initial effect of organic acids and enhance both immediate and residual control of *Salmonella* in meat systems.

### Influence of chitosan and organic acids on *L. monocytogenes* counts

Samples treated with chitosan exhibited consistently lower counts than the control throughout the storage period for both concentrations tested. Other studies have demonstrated chitosan’s ability to reduce *Listeria* counts across different matrices and storage conditions. Shekarforoush *et al*. [50] evaluated the microbial quality of ready-to-barbecue chicken and the growth inhibition of *L. monocytogenes* and *E. coli* O157:H7 inoculated onto samples stored at 3°C, 8°C, and 20°C. These authors reported that chitosan (1 mg/g) did not inhibit aerobic or psychrophilic bacteria, Enterobacteriaceae, or *E. coli*, but was effective against *L. monocytogenes*, with counts approximately 0.5 log lower than those of the control samples after 72 h at 8°C. Paparella *et al*. [51] studied the potential of chitosan against spoilage bacteria and *L. monocytogenes* in fresh pork meat. Pork fillets were inoculated, dipped in 1% chitosan, packed under modified atmosphere conditions (70% O_2_, 20% CO_2_, and 10% N_2_), and stored at 4°C for 15 days. A 2 log decrease was obtained after 2 days of storage in samples treated with chitosan. In addition, the treatment extended the shelf life of the meat by 8 days compared with the control samples. Similar findings were reported by Economou *et al*. [52], who evaluated chitosan coatings applied to raw beef and mutton cuts inoculated with *L. monocytogenes* and observed significant reductions in *L. monocytogenes* counts during refrigerated storage. These results support chitosan’s role as an effective antimicrobial agent in meat matrices and corroborate the sustained inhibitory effect observed in the present study on pig skin.

The antimicrobial activity of chitosan appears to be affected by several factors; however, the exact mechanism of antibacterial activity has not yet been fully elucidated [35]. Electrostatic interactions between the polycationic structure of chitosan and anionic groups on the bacterial cell surface may disturb the cell wall of Gram-positive bacteria or the outer membrane of Gram-negative bacteria. These interactions can increase cytoplasmic membrane permeability, leading to the loss of essential constituents, such as enzymes, nucleotides, and ions [25, 53]. In addition, chitosan can chelate essential metal ions and interfere with microbial metabolism, further contributing to its inhibitory activity [53]. These mechanisms are influenced by several parameters, including pH, molecular weight, degree of deacetylation, and the physical state of chitosan [25, 53]. The present results showed that 0.5% chitosan was sufficient to control bacterial proliferation for up to 48 h, whereas 0.2% chitosan exerted a milder inhibitory effect. This suggests that, at lower concentrations, the availability of positively charged amino groups may be insufficient to fully destabilize bacterial membranes or maintain persistent antimicrobial activity.

For samples treated with organic acids, both lactic acid and citric acid initially reduced microbial counts compared with the control, although significant increases were observed over time. The results demonstrate that although organic acids provided an immediate reduction in *L. monocytogenes* counts, chitosan, particularly at 0.5%, exerted a more stable and prolonged inhibitory effect. This distinction highlights the potential of chitosan as a sustained antimicrobial agent in meat systems that can limit *Listeria* growth during post-processing storage.

## CONCLUSION

This study demonstrated that both organic acids and chitosan were capable of reducing microbial contamination on pig skin surfaces; however, their effectiveness varied according to the target micro-organism and storage period. For spoilage and hygiene indicator micro-organisms, including mesophilic bacteria, psychrotrophic bacteria, and Enterobacteriaceae, all treatments produced an initial reduction in microbial counts, with the greatest effects generally observed for 5% lactic acid, 5% citric acid, and 0.5% chitosan. Nevertheless, microbial populations increased during refrigerated storage, reducing the differences among treatments over time.

For foodborne pathogens, distinct antimicrobial patterns were observed. Organic acids resulted in an immediate reduction in *Salmonella* and *L. monocytogenes* counts but primarily exerted a bacteriostatic effect during storage. In contrast, chitosan demonstrated a more sustained antimicrobial activity. The 0.5% chitosan treatment resulted in a progressive reduction of *Salmonella* counts, achieving a decrease of 1.46 log CFU/cm² over 48 h and maintaining counts 2.68 log CFU/cm² lower than those of the control group. Similarly, chitosan effectively controlled the proliferation of *L. monocytogenes*, with the 0.5% treatment limiting bacterial growth to only 0.44 log CFU/cm² over 48 h and maintaining counts 2.66 log CFU/cm² lower than those of untreated samples.

From a practical perspective, these findings indicate that chitosan may represent a promising natural alternative or complementary intervention to conventional organic acid-based decontamination strategies in pork production systems. Its ability to maintain prolonged antimicrobial activity during refrigerated storage may improve the microbiological safety and quality of carcasses, thereby supporting efforts to reduce pathogen transmission along the food chain within a One Health framework.

A major strength of this study is the direct comparison of chitosan and organic acids under standardized experimental conditions using pig skin, a matrix closely associated with carcass contamination during slaughter operations. Furthermore, the simultaneous evaluation of spoilage micro-organisms and major foodborne pathogens provides a comprehensive assessment of treatment efficacy. However, several limitations should be acknowledged. The study was conducted under controlled laboratory conditions using experimentally inoculated pig skin samples and a relatively short storage period of 48 h. Therefore, the results may not fully represent the complexity of commercial slaughterhouse environments, natural contamination patterns, or extended storage conditions.

Future studies should evaluate the application of chitosan on whole carcasses under industrial processing conditions, investigate longer storage periods, and assess its efficacy against a broader range of foodborne micro-organisms. Research exploring combinations of chitosan with organic acids or other natural antimicrobials may also help identify synergistic effects that enhance both immediate and long-term microbial control.

Overall, the findings demonstrate that chitosan, particularly at a concentration of 0.5%, exhibits substantial potential as a carcass decontamination strategy. Compared with the organic acids evaluated, chitosan provided more persistent inhibition of *Salmonella* and *L. monocytogenes* during refrigerated storage, supporting its use as a sustainable antimicrobial intervention for improving pork safety and quality.

## DATA AVAILABILITY

All generated data are included in the revised manuscript. Supplementary data and raw datasets are available from the corresponding author.

## AUTHORS’ CONTRIBUTIONS

AE and CS: Conceptualization and study design. MM-A, CS, and MC: Data collection. MC, MM-A, KS, and AE: Literature review. MC, MM-A, KS, CS, and AE: Data analysis and interpretation. MC, MM-A, and CS: Manuscript drafting. MM-A, KS, AE, and CS: Manuscript review and editing. AE: Supervision. All authors have read and approved the final version of the manuscript.
